# Patient comfort in percutaneous coronary interventions

**DOI:** 10.15537/smj.2023.44.5.20220872

**Published:** 2023-05

**Authors:** Buse Çıracı, Selda Rızalar

**Affiliations:** *From the Coronary Angiography Unit (Çıracı), Memorial Bahçelievler Hospital; and from Department of Surgical Nursing (Rızalar), University of Health Science, Hamidiye Nursing Faculty, Istanbul, Turkey.*

**Keywords:** angiography, comfort, coronary, nursing, patient comfort

## Abstract

**Objectives:**

To identify patients’ general comfort levels in percutaneous coronary intervention.

**Methods:**

This descriptive research included 2 hundred cardiac patients, whom were in the Medipol Mega University Hospital, Istanbul, during the period between May 2018 and May 2019. The data were obtained by General Comfort Questionnaire and evaluated using mean, standard deviation, percentage and t-test.

**Results:**

In this study reports that patients had the mean total comfort score as 3.03±0.3. They acquired the maximum score from the psychospiritual comfort subdimension and the minimum score from the physical comfort subdimension. Patients who experienced transradial percutaneous coronaryintervention had statistically higher general, physical, psycospiritual and environmental comfort levels than those who had transfemoral intervention (*p*<0.05). According to both access methods, relief and ease levels were significantly different.

**Conclusion:**

Patients who experienced percutaneous coronary intervention have above medium general comfort levels. Their physical comfort scored lowest within the comfort dimensions investigated in this study. The comfort level of the patients to whom the transradial method was applied was found to be higher in comparison with the transfemoral method.

**C**ardiovascular diseases (CVDs) are among the most important illness affecting human health in our age.^1-3^ Coronary artery diseases are in the first place among cardiovascular diseases, and they are at the forefront among the causes of mortality after the age of 40.^4-6^

According to the evaluations of the World Health Organization, coronary artery disease was observed in 15.8 million people in 2010, and it is predicted that this number will reach 23 million in 2030.^4^ According to the data of the Ministry of Health, the cause of 37% of deaths under the age of 70 in Turkey is cardiovascular diseases.^1,7^ Turkish Statistical Institute’s mortality results show that, the rate of heart diseases in total deaths is gradually increasing.^8^

Coronary angiography (CAG) is the most chosen method in the diagnosis and treatment of coronary artery diseases. Coronary interventions, including angioplasty or stent placement, can also be performed during angiography.^9^

Comfort is used synonymously with the word relief. In nursing, it is a complex multidimensional concept related to overcoming problems with physical, psychospiritual, social, and environmental dimensions and ensuring peace. Comfort is an expected and desired outcome of nursing care. According to Kolcaba K, and Kolcaba R,^10^ comfort is etymologically derived from “confortare” in Latin, which denotes “to reinforce more, strengthen.” In this sense, it means reinforcing, promoting, encouraging, helping, and relieving.^10^

In percutaneous coronary interventions (PCIs), pain in the intervention area, hospitalization requirement, a failure to meet needs such as activity and eating, and experiencing anxiety adversely affect comfort.^11-13^ When health care is needed, comfort is first required to be provided because when comfort is cared, considerably better health care services can be reached. It has been determined that comfort has both physical and mental effects on patients’ experiences in the field of health. The patient’s comfort level is considered an element of the quality of care.^10,14^

While radial or femoral arterial access is used in coronary interventions, the use of radial artery access has become widespread in recent years. Interventions with radial artery access are preferred due to its aspects such as fewer complications at the site of access, early mobilization, early discharge, low cost, and improved quality of life.^11,12,15^ Although many sources emphasize that transradial angiography increases success, reduces the time of procedure, and is preferred by patients, it has also been mentioned that its use is limited due to the length of the procedure and procedural failure.^15^ Furthermore, treatment with transfemoral vascular access reported to bring higher cost of procedures, higher complication rates and longer time spent at hospital.^7,11,12,16^

There are studies involving pain assessment after coronary interventions for nursing care. Although some studies have reported that interventions with radial access increase patient comfort, there are very few studies in which comfort level is evaluated using a comfort scale.^20^ Nowadays, it has become important to perform procedural interventions applied to a large number of patients under the most comfortable conditions. In fact, it has become highly important that patients are adequately informed to choose the most advantageous method. In addition to increasing the patient’s comfort by providing physical care in a multidisciplinary team, nurses can also increase comfort by explaining the benefits and risks of PCI according to access route options. Nurses can help patients decide on the access point.^15^ Anxiety can be reduced by adequately informing the patient, and an independent decision-making process is also supported. This study aimed to identify comfort levels of the patients experiencing PCI, and to determine whether different vascular access methods affect the comfort level.

## Methods

It is a descriptive and cross-sectional study. Research presentation was made in accordance with the Strengthening the Reporting of Observational studies in Epidemiology principles. The study was carried out between May 2018 and May 2019 in the angiography department of a non state university hospital in Istanbul. Since it is usual procedure for patients to schedule their appointments for their angiography several days in advance, they are admitted to the hospital where they receive treatment only a few hours before the procedure. Furthermore, patients hospitalized in the coronary intensive care or cardiology service are also transported to the unit at the time of the procedure. Before the intervention, patients who initially came from their home are first directed to the outpatient service. After they are prepared for the procedure, they are taken to the angiography laboratory, and the procedure is performed. After the intervention is completed, patients are followed up in the outpatient service. Patients’ general condition, vital signs, electrocardiography, local pain and bleeding, circulation and movement of the extremity are monitored for allergic reactions. Unless there is an unusual condition, patients are discharged after receiving training 2 hours after radial intervention and 6 hours after the femoral intervention.

The patients who experienced PCI in the angiography labratory of a foundation university hospital constituted the research population. The 200 cardiac patients who underwent intervention between the specified dates, meet the sample selection criteria and consented to join in the research study constituted the sample. In the calculation of the study’s sample size, an appropriate sample size was found to be 200 with an effect size of 0.5, an alpha error of 0.05 (95% confidence level), and a power of 0.91.

Patients who were oriented to time and place, could speak Turkish, were aged 18 years and over, had normal vital signs, volunteered to participate in the study, and had undergone PCI including one or several of angiography, angioplasty, or stent interventions were included in the sample. Individuals with hearing loss, psychiatric and mental illnesses were not included in the sample. Individuals having problems related to hearing loss, psychiatric or mental illnesses were excluded from the sample.

Data collection was carried out using Patient characteristics form and the GCQ (General Comfort Scale) in a face-to-face interview. The general comfort scale used in the present study was developed by Kolcaba^17^ in 1992. It consists of 4 subscales and 3 levels to evaluate the patient’s comfort status and determine comfort-related nursing services and comfort-related needs. The subscales of the questionnaire consist of a total of 48 items, including ease (16 of 48), relief (17 of 48), and transcendence (15 of 48).

The scale is evaluated on a 4-point Likert type. The minimum and maximum (min-max) scores to be acquired from the scale are 48 min and 192 max. The mean score value of the scale is calculated between 1-4 by dividing it by the number of items. Cronbach’s alpha value of the questionnaire was 0.88 in Kolcaba’s study,^18^ 0.85 in the Turkish^19^ version, and 0.78 in this study. After the patient was admitted to the clinic, an explanation was provided, and permission was obtained for the study, and the patient characteristics form was filled out. The comfort questionnaire was filled out before the patient left the outpatient service. Data collection was held by interviewing the patients face-to-face before they were discharged, in approximately 15 minutes.

The ethics committee approval of Istanbul Medipol University’s non-interventional ethics committee and the institutional permission of Medipol Mega Hospital were obtained before starting the application (274/2016). By having the participants sign an informed consent form, it was confirmed that they participated in the study voluntarily, without any pressure or coercion. Permission to use the GCQ was obtained from the author who performed its adaptation study. All procedures in this study were in conformity with the Declaration of Helsinki of 1975, as updated in 2013.

### Statistical analyses

The data in this study were analyzed using the SPSS Statistics for Windows, version 25 (IBM Corp., Armonk, N.Y., USA). Descriptive statistics (number, percentage, mean, standard deviation, min-max) were used to identify demographic and health-related characteristics of patients undergoing PCI. All of the comfort variables, which are the subscales of the GCQ, were identified by taking the average of the items constituting these variables. Kolmogorov-Smirnov test was used to evaluate the conformity of data to normal distribution. Then, the independent sample t-test, one of the parametric tests, was used to evaluate differences in comfort scores according to vascular access methods. A statistical significance level was considered as *p*<0.05.

## Results

Individual and disease-related characteristics of patients are presented in detail in Table 1. Approximately half of the patients had their first angiography, and approximately half of them had one or more chronic diseases. The intervention was performed as an outpatient and elective procedure in 82% of them. Of the patients, 69% and 20% perceived their disease as a treatable disease and a manageable disease, respectively. Of the individuals, 35% and 55% perceived their disease as very serious and moderately serious, respectively. While 81% of the patients were independent enough to be self-sufficient in activities of daily living, 14% of them were semi-dependent. Angiography was performed from the radial artery in 67% of the patients and the femoral artery in 33% of the patients.

**Table 1:** - Patients’ characteristics and disease details.

Characteristics	n	%	Disease details of the patients	n	%
***Gender***			***Chronic disease***		
Woman	52	26	None	95	48
Men	148	74	One	57	29
***Age***			More than one	48	24
20-39 year	13	7	***Intervention***		
40-59 year	93	46	Angiography	126	63
>60y	94	47	Angioplasty	9	5
Body mass index			Stend	65	33
Normal	30	15	***Intervention site***		
Overweight	98	49	Transradial	134	67
Obese	72	36	Transfemoral	66	33
***Marital status***			***Arrival at the hospital***		
Married	185	93	From home	164	82
Single	15	8	From hospital	36	18
***Education***			***Urgency of intervention***		
Illiterate	9	5	Urgent	36	18
Lliterate	10	5	Elective	164	82
Primary school	119	60	***Number of ıntervention***		
Lise	32	16	First	99	50
University	30	15	More than one times	101	51
***Employment***			***Severity of disease***		
Employed	67	34	Serious	70	35
No	133	67	Moderately serious	110	55
***Insurance***			Not serious	20	10
Private	8	4	***Prognosis***		
Social security	140	70	Curable	137	69
Other	52	26	Manageable	55	28
***Income***			Getting worse	8	4
Low	23	12	***Living activities***		
Middle	147	74	Dependent	12	6
High	30	15	Semi-dependent	27	14
			Independent	161	81

The patients’ mean scores of the GCQ subscales and levels are presented in Table 2. In our study, among the comfort subscales, the mean score of the physical comfort subscale was 2.89±0.41, of the psychospiritual comfort subscale was 3.21±0.42, of the environmental comfort subscale was 2.92±0.45, and of the sociocultural comfort subscale was 2.97±0.35. The mean score of general comfort was found to be 3.00±0.3. The mean scores of comfort levels were found to be 3.02±0.35 for ease, 3.01±0.36 for relief, and 2.96±0.38 for transcendence.

**Table 2:** - Patients’ mean scores of the General Comfort Questionnaire dimensions and levels.

Questionnaire dimensions and levels	Mean score	Min-Max
General comfort	3.00±0.30	2.2 - 3.7
***Comfort dimensions***		
Physical comfort	2.89±0.41	1.5 - 4.0
Psychospiritual comfort	3.21±0.42	1.9 - 4.0
Environmental comfort	2.92±0.45	1.8 - 3.8
Sociocultural comfort	2.97±0.35	1.8 - 3.8
***Comfort levels***		
Ease	3.02±0.35	1.9 - 3.8
Relief	3.01±0.36	2.1 - 3.8
Transcendence	2.96±0.38	1.5 - 3.7

In Table 3, the mean scores of general comfort and physical, psychospiritual, environmental, and sociocultural comfort, which are the subdimensions of general comfort, and the mean scores of ease, relief and transcendence, which are comfort levels, were compared according to the vascular access method in angiography. According to the intervention method, a statistically significant difference was detected between the mean scores of physical, psychospiritual, environmental and general comfort and ease and relief (*p*<0.05).

**Table 3:** - General Comfort Questionnaire total, dimension and level score averages by percutan coronary interventions access method.

Comfort Questionnaire Dimensions and levels	Access method		Test and *p*-value
	Transradial (n=134)	Transfemoral (n=66)	
	Mean score	Mean score	t ; p
General comfort	3.11± 0.29	2.98± 0.31	3.113; 0.002
***Dimensions***			
Physical comfort	3.06 ± 0.41	2.86± 0.40	3.162; 0.002
Psychospiritual comfort	3.35± 0.43	3.21 ± 0.41	2.364 0.019
Environmental comfort	3.01± 0.45	2.85± 0.43	3.739; 0.000
Sociocultural comfort	2.96± 0.34	2.99 ±0.37	-0.488; 0.626
***Levels***			
Ease	3,. 2 ± 0.33	3.01 ± 0.37	2.137 ; 0.034
Relief	3.12± 0.34	3.00 ± 0.39	2.187; 0.030
Transcendence	2.98 ±0.38	2.92 ± 0.36	1.28; 0.202

When the scores were examined, it was seen that patients who underwent transradial intervention had higher average scores of physical, psychospiritual, environmental, and general comfort compared to the transfemoral access group (*p*<0.05).

Furthermore, ease and relief levels of the patients who underwent transradial intervention scored higher than those who underwent transfemoral intervention. Of note, the mean scores of the transcendence levels of the patients who underwent transradial intervention and those who underwent transfemoral intervention did not statistically differ (*p>0.05).*

## Discussion

The data obtained in this study, conducted to investigate the comfort levels of patients in transradial and transfemoral vascular access interventions, were discussed based on the literature.

In this study, the average score of the GCQ was found to be 3.00±0.3. The mean scores of the comfort subscale and level varied between 2.89±0.41 and 3.21±0.42, the minimum mean score was in the physical comfort subscale, and the maximum mean score was in the psychospiritual comfort subscale. The comfort level and subscale scores were close to each other in patients who underwent angiography and vascular interventions, and the comfort level was below excellent but above the average.

Although being in limited numbers, there are studies which were conducted in Turkey and used the GCQ in patients with PCI. Çakır^20^ (2019) compared the comfort levels before and after the intervention in 107 patients who underwent transradial angiography. It was determined that the patients’ general comfort scores were higher after the intervention compared to those before the intervention.^20^ There were some studies in which the comfort level was evaluated in medical surgical patients. In the study conducted by Anuş Topdemir (2019), the mean score of the GCQ at the third postoperative hour was found to be 2.78±0.32 in patients who underwent surgery,^21^ 3.09 in uremic patients22, 2.79±0.34 in patients with diabetes,^23^ and 2.86 in older adults.^24^ It can be said that the general comfort level in our study sample was similar to those in other studies. According to the comfort theory, nurses deal with 3 levels of comfort while helping to meet human needs. The statements for the levels indicate the details on patient comfort and altogether show the holistic structure of the nursing practice. Comfort is in a dynamic state in this conceptualization and may change rapidly, either positively or negatively. The level of comfort is higher when pain is absent. Improving comfort may also reduce the patient’s anxiety-related complaints by increasing hope and confidence. Kolcaba reported that improving comfort increased patient and nurse satisfaction, provided early discharge, decreased the rehospitalization rate, and reduced costs.^10,18^ In his study, Reynolds^25^ (2001) reported that raising the head of the bed, changing the position, back massage, and early ambulation increased comfort. In the study carried out by Tongsa and Thamlikitkul^26^ (2012), it was determined that early mobilization after PCI increased comfort, shortened the length of stay, and reduced costs.

In this study, the mean scores of comfort levels were found to be 3.02±0.35 for ease, 3.01± 0.36 for relief, and 2.96±0.38 for transcendence. In a more recent study, Kara and Işık Andsoy^27^ (2018) have examined the impact of education delivered to participants prior to pilonidal sinus surgery on comfort, and have reported the comfort scores of 1.94±0.38 for ease, 2.29±0.36 for relief, and 2.56±0.22 for transcendence during the surgical process.

Ease is felt by an individual when he gets rid of problems as a result of satisfaction, alleviating anxiety, and meeting the needs, and it is required to return to normal functions. Relief refers to meeting the needs, eliminating discomfort, being at peace, and being self-satisfied.^10,18^ Transcendence is a state of comfort when patients can overcome difficulties. Its aim is to ensure that individuals can overcome their problems and are free to control and plan their destiny at a certain time and in a certain situation according to their potential. Transcendence level can be reached only when a person’s comfort needs are fully met, which is the superiority of comfort.^10,18^

All 3 comfort levels positively affect the patient’s performance and are theoretically energizing components.^28^

In our study, when all comfort subscales were considered, it was observed that the physical comfort level was the lowest in the patients. Another study by Huant et al^29^ examined the effect of preoperative positioning on postoperative pain and discomfort in patients and found that patients had the highest psychospiritual comfort score and the lowest physical comfort score on the first postoperative day.

In a study by Wang et al^30^ examined the effect of mindfulness in the rehabilitation of stroke patients. The psychospiritual comfort score was found to be the highest, and the physical comfort score was found to be the lowest.30 The results of our study are appropriate with the literature. Physical comfort is related to bodily perceptions. Fluid electrolyte balance, blood biochemistry, oxygen saturation, and metabolic functions affect physical comfort. According to Kolcaba, when there is an abnormality in physiological indicators, the concept of comfort will be adversely affected.18,31 Pain is one of the most important factors reducing physical comfort.18 In the study performed by Çarık (2020), it was determined that the fear of pain before the operation affected the comfort levels of patients after the operation.32 Angiography is a painful procedure. Low physical comfort due to pain is an expected situation. On the other hand, psychospiritual comfort consists of mental, emotional, and spiritual components. Since the procedure is performed in a short time in patients undergoing percutaneous intervention, the score obtained in this subscale may have been found to be higher. Anxiety is an important factor that can reduce psychospiritual comfort in these patients.^28^ Care interventions that can increase comfort may include making tactile contact and encouraging patients to use their own relief methods to find spiritual peace.^18^ Environmental comfort includes external factors such as heat, light, noise, color, and their effects on people. Nowadays, it is known that environmental comfort should be provided to support the physical and mental functions of an individual.^10,18^ In our study, the average score of environmental comfort was specified to be 2.91±0.4, which was close to the physical comfort score and suggested that perfect environmental conditions could not be achieved.

In this study, the intervention was performed by providing transradial access in 67% of the patients and transfemoral access in 33% of them. A significant difference was found between the general comfort levels of the patients who underwent radial and femoral interventions (*p*<0.05). Upon examining the mean scores, it was revealed that the physical, psychospiritual, environmental, and general comfort levels of those who underwent transradial intervention were higher than those with transfemoral interventions. Furthermore, it was examined that the ease and relief scores of the patients who underwent transradial intervention were higher. While the chance of success and patient comfort increase in interventions performed by transradial access, the time of procedure is shortened, and complication rates are reduced. The risk of developing ischemia in the hand decreases due to the double blood supply.^11,33,34^ A study carried out to determine the effects of a vascular access method on patient comfort revealed that patients with the radial intervention felt more at ease and more comfortable and patients who underwent transfemoral intervention complained more on inactivity, defecation, micturition, and sleep.^13^ In a study carried out with nurses responsible for post-procedure care in percutaneous coronary interventions, nurses indicated that radial access was more comfortable, less embarrassing and less complicated, patients were discharged early, and care was easier. A study carried out in Lahore in 2021 determined that those with post-angiography femoral access experienced more local pain and more discomfort compared to those with radial access.^15^ In the study performed by Fens^12^ (2015) in the Netherlands, patients who underwent both radial and femoral interventions were questioned, 2 vascular access routes were compared based on the patient’s perspective, and no access route was found to be superior. It was indicated that the vascular access decision was a preference-sensitive decision and that the importance of the procedure’s features might vary according to the patient.^12^ It was indicated that joint decision-making with healthcare professionals and patients might contribute to patient-centered care.

In the study examining the satisfaction of patients, patients’ families, and nurses with vascular access, it was determined that nurses were mostly more satisfied with the radial access, followed by patients and their relatives.^35^ It was found that the majority of the patients and their relatives had insufficient information regarding the types of vascular access. According to patient references in the study carried out by Wilcoxson,^36^ it was determined that early mobilization increased comfort. Another study managed by Louvard et al^37^ (2001) reported that patients from transradial group had higher comfort levels.

It was indicated that the majority of the patients (58%) who experienced both interventions preferred the radial intervention.^37^ Kok et al^38^ have studied the vascular access preference of patients, and showed that 71.1% of the patients who had experienced both interventions preferred the transradial intervention.

Taken together, our study unravels parallel results to the literature, with the consistent preference of the patients for transradial percutaneous coronary intervention rather than transfemoral intervention.

### Study limitations

A main limitation in this study is the sample size. Since the results of the study can only be generalized to the research sample, generalizing the results to all PCI patients may not be applicable. Thus, it is recommended to design similar studies with larger groups and different samples.

In conclusion, the general comfort levels of Turkish patients undergoing PCI are above the average, but the physical comfort and environmental comfort levels are lower than the others. Comfort level is higher in transradial procedures compared to transfemoral procedures. Nurses can improve comfort by providing education and care to their patients. In particular, explaining the advantages and disadvantages of vascular access methods for care may support the patient’s decision-making in the preference process.

## References

[1] Ministry of Health Publication No: 988. Turkey Cardiovascular Diseases Prevention and Control Program. Action Plan (2015-2020). Ankara, T.C.. [Updated 2020; Accessed 2022 Oct]. Available from: http://www.heartcharter.eu/download/English.pdf

[2] Karadakovan A , Aslan FE . Medical Surgical Nursing Care, 3rd Ed. Academician Medical Publication Göktuğ Press, Ankara, Turkey; 2017.

[3] Murphy JG , Lloyd MA . Mayo Clinic Cardiology: Concise Textbook, 4th ed. Oxford University Press, New York (NY). 2015.

[4] World Health Organization. Preventing chronic disease: A Vital Investment. [Updated 2021; Accessed 2023 April]. Available from: https://apps.who.int/iris/handle/10665/43314

[5] Nixon (lan) JV . Translate Editors: Kozan Ö, Keleş İ, AHA (American Hard Association) Cardiac Council, 3rd Edition, 2014.

[6] Balbay Y , Bener S , Kaygusuz T , Çay S , İlkay E. [Coronary revascularization (World and Türkiye examples)]. Turk Kardiyol Dern Ars 2014; 42: 245–252. 24769816 10.5543/tkda.2014.49765

[7] Mann T , Cowper PA , Peterson ED , Cubeddu G , Bowen J , Giron L . Transradial coronary stenting: comparison with femoral access closed with an arterial suture device. Catheter Cardiovasc Interv 2000; 49: 150–156. 10642762 10.1002/(sici)1522-726x(200002)49:2<150::aid-ccd7>3.0.co;2-f

[8] Turkey Statistical Institute. Death Causes Statistics, 2018. [Updated 2018; accessed 2019 May]. Available from: https://data.tuik.gov.tr/Bulten/Index?p=Olum-Nedeni-Istatistikleri-2018-30626#:~:text=%C3%96l%C3%BCm%20vakalar%C4%B1n%C4%B1n%20%38%2C4’,ile%20solunum%20sistemi%20hastal%C4%B1klar%C4%B1%20izledi.&text=%C4%B0skemik%20kalp%20hastal%C4%B1%C4%9F%C4%B1ndan%20sonra%20s%C4%B1ras%C4%B1yla,3%20ile%20hipertansif%20hastal%C4%B1klar%20g%C3%B6r%C3%BCld%C3%BC

[9] Thomson PD . Preventive Cardiology. Topol E. (Eds) in Textbook of Cardiovascular Medicine. 1.Ed İstanbul: Düzey Publication; 2005. p. 1–257.

[10] Kolcaba K , Kolcaba R . An analysis of the concept of comfort. J Adv Nurs 1991; 16: 1301–1310. 1753026 10.1111/j.1365-2648.1991.tb01558.x

[11] Anjum I , Khan M , Aadil M , Faraz A , Farooqui, M , Hashmi A , et al. Transradial vs. transfemoral approach in cardiac catheterization: a literature review. Cureus 2017; 9: e1309. 28690943 10.7759/cureus.1309 PMC5493462

[12] Fens AL . Patient Preferences for Radial versus Femoral Vascular Access Options by Coronary Angiography and Intervention (PREVAS). University of Twente, School of Management and Governance, Department of Health Technology and Services Research. Enschede, the Netherlands; 2015.

[13] Gürses B . Evaluation of transradial and transfemoral angioplasty applications in terms of patient comfort. Surgical Diseases Nursing Master Thesis, İstanbul, Turkey; 2017.

[14] Erdemir F , Çırlak A . Comfort Concept and Use in Nursing. Deuhyo Ed 2013; 6: 224–230.

[15] Tasneem S , Nazia Ilyas N . Levels of Pain in Patients Undergoing Coronary Invasive Procedures in Trans-Radial Versus Trans- Femoral Approaches: A Cross-Sectional Study. MNJ 2018; 10: 26–34.

[16] Jamil O , Chandra, Shukla P , Contractor S , Kumar A . Radial versus femoral access: is there a preference among nursing care providers? Scientific e-Posters 2019; 30: 247–248.

[17] Kolcaba K . Holistic comfort: Operationalizing the construct as a nurse-sensitive outcome. ANS Adv Nurs Sci 1992; 1: 1–10. 10.1097/00012272-199209000-00003 1519906

[18] Kolcaba K . Comfort theory and practice. a vision for holistic health care and research. Clin Nurse Spec 2005; 19: 49.

[19] Kuğuoğlu S , Karabacak Ü . Adaptation of general comfort scale to Turkish. Florence Nightingale J Nurs 2008; 61: 16–23.

[20] Çakır E . Comparison the levels of comfort and anxiety related to radial artery catheterization, İstanbul Okan University, Health Science Institute, 2019.

[21] Anuş Topdemir E . The Effect of Acupressure and Reiki Administration after Laparoscopic Cholecystectomy on the Pain and Comfort Levels of Patients, Inönü University, Nursing Doctora Thesis, Malatya, Turkey, 2019.

[22] Çalışkan T , Çınar PS . Does itching affect comfort in uremic patients receiving or not receiving hemodialysis treatment? Türk Nefroloji, Diyaliz ve Transplantasyon Hemşireleri Derneği, Nefrology Nursing Journal 2019;14: 84–96.

[23] Özden G . The effect of accepting the disease on the comfort level in patients with type 2 diabetes mellitus. İnönü Üniversity, Health Science Institute Master thesis, 2018.

[24] Göke G , Çınar Yücel Ş . The effect of back massage and hand massage on comfort and anxiety in elderly people living in nursing homes. Ege University Institute of Health Sciences, Department of Nursing Fundamentals, 2016.

[25] Reynolds S , Waterhouse K , Miller K . Patient care after percutaneous transluminal coronary angioplasty. Dimens Crit Care Nurs 2001; 20: 44–51. 10.1097/00003465-200105000-00013 22076400

[26] Tongsai S , Thamlikitkul V . The safety of early versus late ambulation in the management of patients after percutaneous coronary interventions: a meta-analysis. Int J Nurs Stud 2012; 49: 1084–1090. 22521852 10.1016/j.ijnurstu.2012.03.012

[27] Kara M , Işık Andsoy . I The Effects of Preoperative Education Before Pilonidal Sinus Surgery on Patient’s Anxiety and Comfort, Health Sciences Profession 2018; 5(3): 397–403.

[28] Wilson L , Kolcaba K . Practical Application of Comfort Theory In The Perianesthesia Setting. J Perianesth Nurs 2004; 62: 164–173. 10.1016/j.jopan.2004.03.006 15195275

[29] Huang J , Zhang D , Li SJ , Xi Y , Cui LY , Gao FL . Preoperative practice of surgical position reduces postoperative pain and discomfort in patients receiving kidney surgeries: a nonrandomized pilot study. Ther Clin Risk Manag 2018; 14: 1111–1114. 29942133 10.2147/TCRM.S152836 PMC6005319

[30] Wang M , Liao W , Chen X . Effects of a short-term mindfulness- based intervention on comfort of stroke survivors undergoing inpatient rehabilitation. Rehabil Nurs 2019; 44: 78–86. 30830883 10.1097/rnj.0000000000000098

[31] Kolcaba K , Dimarco MA . Comfort Theory and its application to pediatric nursing. Pediatr Nurs 2005; 31: 187–194. 16060582

[32] Çarık S . The effect of pre-operational pain fear on post- operative pain and general comfort, Istinye University, Surgical nursing department, Istanbul, 2020.

[33] Ferrante G , Rao SV , Jüni P . Radial versus femoral access for coronary interventions across the entire spectrum of patients with coronary artery disease: a meta-analysis of randomized trials. JACC Cardiovasc Interv 2016; 9: 1419–1434. 27372195 10.1016/j.jcin.2016.04.014

[34] Kanei Y , Kwan T , Nakra NC . Transradial cardiac catheterization: a review of access site complications. Catheter Cardiovasc Interv 2011; 78: 840–846. 21567879 10.1002/ccd.22978

[35] Abd-Elmaged ES , Mohammed GT , Abd-algelil A . Radial Versus Femoral Access For Coronary Angiography or Intervention And The Effect on The Nurses, Patients And Relatives’ Satisfaction. IOSR-JNHS 2018; 7: 17–27.

[36] Wilcoxson VL . Early ambulation after diagnostic cardiac catheterization via femoral artery access. J Nurse Pract 2012; 8: 810–815.

[37] Louvard YV , Lefe`vre TH , Armelle A , Marie-Claude, M . Coronary angiography through the radial or the femoral approach. Catheter Cardiovasc Interv 2001; 52: 181–187. 11170325 10.1002/1522-726x(200102)52:2<181::aid-ccd1044>3.0.co;2-g

[38] Kok MM , Weernink MGM , Birgelen C , Fens A , Heijden LC , Til JA . Patient preference for radial versus femoral vascular access for elective coronary procedures: The PREVAS study. Catheter Cardiovasc Interv 2018; 91: 17–24. 28470994 10.1002/ccd.27039 PMC5811812

